# Postoperative management of patients undergoing cardiac surgery in Austria: A national survey on current clinical practice in hemodynamic monitoring and postoperative management

**DOI:** 10.1007/s00508-018-1403-3

**Published:** 2018-10-29

**Authors:** Johannes Menger, Maximilian Edlinger-Stanger, Martin Dworschak, Barbara Steinlechner

**Affiliations:** Division of Cardiac Thoracic Vascular Anaesthesia and Intensive Care Medicine, Department of Anaesthesia, Intensive Care Medicine and Pain Medicine, Medical University of Vienna, Spitalgasse 23, 1090 Vienna, Austria

**Keywords:** Survey, Cardiac surgery, Hemodynamic monitoring, Inotropic drugs, Volume therapy

## Abstract

**Background:**

No data are currently available regarding the current clinical practice in postoperative care of cardiac surgical patients in Austria.

**Objective:**

The study investigated the current intensive care management concerning hemodynamic monitoring and strategies to treat common perioperative disorders of patients after cardiac surgery in Austria.

**Methods:**

A survey consisting of 31 questions was sent to intensivists at all 9 hospitals offering cardiac surgery in Austria.

**Results:**

The response rate was 100%. The mean number of procedures on cardiopulmonary bypass per centre was 722 ± 223. In the majority of cases postoperative critical care is performed by anesthesiologists. Blood gas analysis, pulse oximetry, electrocardiogram, temperature, central venous pressure, arterial pressure and hourly urine output are de facto standard monitoring in all centers. Transesophageal echocardiography is available in all centers and is frequently used. Crystalloids are the first choice for volume replacement, whereas levosimendan and adrenaline are employed for the treatment of low cardiac output syndrome.

**Conclusions:**

This study provides insights into the current state of postoperative management of cardiac surgical patients in Austria. Standard monitoring as proposed by international guidelines is well established in Austrian intensive care units. Echocardiography is widely seen as a very important tool in the postoperative care of cardiac surgical patients. Knowledge about the status quo of postoperative intensive care management of cardiac surgical patients enables further development of patient care.

**Electronic supplementary material:**

The online version of this article (10.1007/s00508-018-1403-3) contains supplementary material, which is available to authorized users.

## Introduction

There are nine centers in Austria serving patients requiring cardiac surgery. Postoperative care of the great majority of these patients is provided on intensive care units (ICU). Perioperative disorders in patients undergoing cardiac surgery often evolve very dynamically and need immediate response. A prerequisite is, however, that these disorders are promptly detected to be able to respond. Adequate monitoring of hemodynamic parameters and suitable treatment strategies are considered to be key factors in high quality care of patients after cardiac surgery. Common treatment strategies comprise the differentiated use of inotropes, vasopressors and adequate fluid management [1]. The technological progress during the last decades gave the critical care physicians multiple different methods and devices for hemodynamic monitoring to choose from. The choice also includes more sophisticated hemodynamic monitoring devices measuring multiple hemodynamic parameters; this includes pulmonary artery catheter (PAC), transesophageal echocardiography (TEE) and newer less invasive devices.

International guidelines provide relatively non-specific recommendations concerning hemodynamic monitoring and management of patients after cardiac surgery, particularly with respect to hemodynamic and clinical endpoints [1, 2]. Therefore, based on these published guidelines, highly divergent strategies may be employed in the management of the postoperative cardiac surgical patient. Indeed, Italian and German studies reported considerable variations in the postoperative care after cardiac surgery among different institutions [3–5]. Moreover, there is an on-going scientific debate about the appropriate type of fluids for volume replacement in the critically ill [6].

Prompted by the German report in 2005 [4], the German Society of Anesthesiology and Intensive Care Medicine and the German Society of Thoracic and Cardiovascular Surgery published clinical practice guidelines for the postoperative care of the adult cardiac surgical patient [7]. These guidelines have been updated in 2010 [8] and 2018 [1]. So far, no data have been published of the clinical management of cardiac surgery patients in Austrian intensive care units.

The aim of this study was to collect information on the current clinical practice regarding postoperative critical care of these patients in Austria. In particular, this survey intended to address hemodynamic monitoring and management of common postoperative problems.

## Methods

This study was a national survey using a questionnaire. It was moderated by the Austrian Society of Anesthesiology, Resuscitation and Intensive Care (ÖGARI) working group for Cardiothoracic and Vascular Anesthesia (ARGE Herz/Thorax/Gefäß-Anästhesie). Due to the design of the study, approval from the local ethics committee was waived. The survey was e‑mailed in March 2017 to member anesthesiologists at all nine hospitals providing cardiac surgery in Austria. In 2016 cardiac surgery was performed in Graz, Innsbruck, Klagenfurt, Linz, Salzburg, Sankt Pölten, Wels and two centers in Vienna (Krankenhaus Hietzing and Allgemeines Krankenhaus). The participants were asked to complete the questionnaire regarding the practice at their centre during the year 2016 on the primary ICU responsible for cardiac surgical patients.

The template of the questionnaire has already been used in national surveys in Germany in 2005 and 2011 [3, 4] and slightly altered in Italy in 2013 [5]. The questionnaire consisted of 31 questions covering different aspects of perioperative care in adult patients undergoing cardiac surgery. Main aspects of this questionnaire are on hemodynamic monitoring and strategies in the use of drugs and fluids in common disorders in patients after cardiac surgery. The questionnaire was in German and can be found in the Electronic Supplementary Material. For some questions multiple selections could be made.

Final analysis was done after all questionnaires were returned. Only descriptive statistics were used for the analysis of the collected data. Data are presented as a percentage of all Austrian centers offering cardiac surgery. Missing data are also reported for every item whenever this occurred.

## Results

All nine centres (100%) returned the questionnaire by September 2017. Data from one center were incomplete regarding the volume of performed cases. With the exception of this single questionnaire there were no missing data. All institutions were public hospitals and 67% were university hospitals. Structural data of the participating centres are shown in Table 1. Isolated coronary artery bypass graft (CABG) was the most common procedure performed accounting for 31% (2346/7485) of all reported procedures among 8 reporting centers, followed by combined procedures, such as multiple valve surgery/valve surgery plus CABG (19%), aortic valve surgery (19%) and mitral valve surgery (8%). An average of 722 ± 223 procedures were performed on cardiopulmonary bypass in adults at each of the 8 reporting centers in 2016. A heart transplant program was active in 3 centers in 2016 (Graz, Innsbruck and Vienna—Allgemeines Krankenhaus). Of the 8 reporting cardiac surgery centers 2 routinely cared for pediatric patients in 2016 (Linz and Vienna—Allgemeines Krankenhaus).

**Table 1:** Structural data of centers offering cardiac surgery to patients in Austria in 2016

		Number of centers (*N*)
Number of cardiac surgical procedures for cardiopulmonary bypass per year (centers)	<500	2
	500–750	3
	750–1000	2 (3)^a^
	>1000	1
Specialists mainly effectuating postoperative critical care (centers)	Anesthesiologists	8 (89%)
	Anesthesiologists and surgeons	1 (11%)
Department that manages postoperative intensive care unit (centers)	Anesthesiology	8 (89%)
	Surgery	1 (11%)
Intensive care unit dedicated specially to cardiac surgery patients (centers)		3 (33%)

^a^One answer is missing. Missing center has older publicly available data ranging from 750 to 1000 procedures on cardiopulmonary bypass per year in the decade 2000–2009 [25, 26]

**Fig. 1:**
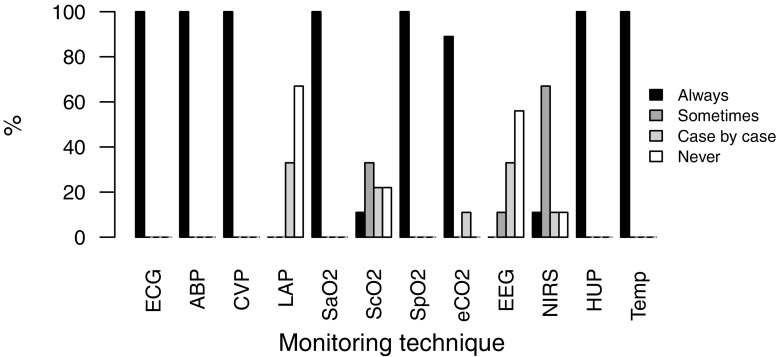
Routine use of postoperative monitoring. *ECG* continuous electrocardiogram, *ABP* arterial blood pressure, *CVP* central venous pressure, *LAP* left atrial pressure, *SaO*_*2*_ arterial oxygen saturation, *ScO2* central venous oxygen saturation, *SpO2* peripheral oxygen saturation, *EEG* processed electroencephalogram, *NIRS* near-infrared spectroscopy, *HUP* hourly urine portions, *Temp* body temperature

Blood gas analysis, pulse oximetry, electrocardiogram, patient temperature, central venous and arterial pressure and hourly urine output were monitored in all nine centres. The use of postoperative monitoring is shown in Fig. 1.

**Fig. 2:**
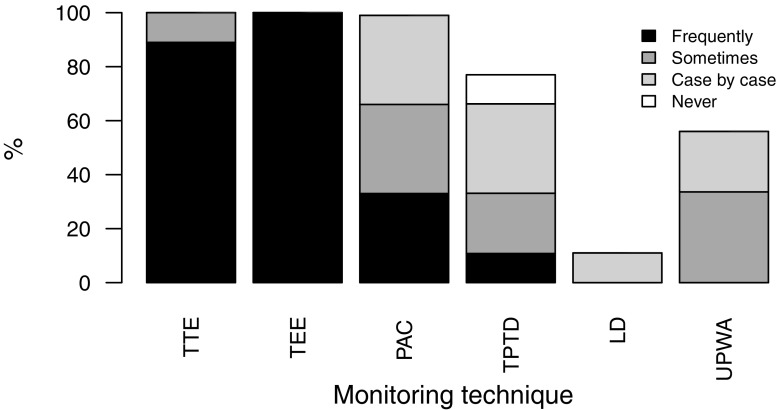
Availability of advanced hemodynamic monitoring and usage. *TTE* transthoracic echocardiography, *TEE* transesophageal echocardiography, *PAC* pulmonary artery catheter, *TPTD* transpulmonary thermodilution, *LD* lithium dilution cardiac output measurement, *UPWA* uncalibrated pulse wave analysis

Pulmonary artery catheters (PAC) are available in all centers. Of the centers three reported frequent use of PAC, three hospitals occasional and another three centers isolated use. Of the centers six preferred continuous and three centers bolus thermodilution cardiac output measurements with PAC. Among the three most important reasons for the usage of PAC were monitoring of pulmonary hypertension (89%), measurement of cardiac output (78%) and monitoring of hemodynamic instability (67%). Of the centers four reported having a specific threshold for the treatment of systolic pulmonary artery pressure (i. e. >50 ± 10 mm Hg) and three centers a threshold for left ventricular ejection fraction (i. e. <32 ± 3%) that necessitate the use of PAC. Availability and usage of advanced hemodynamic monitoring techniques are given in Fig. 2.

All centers employ transesophageal echocardiography (TEE) and have 24/7 in-house availability of a physician experienced with this technique. Among the three most important reasons for the postoperative use of TEE were: hemodynamic instability (100%), suspected cardiac tamponade (100%), and evaluation of valve function (78%).

**Fig. 3:**
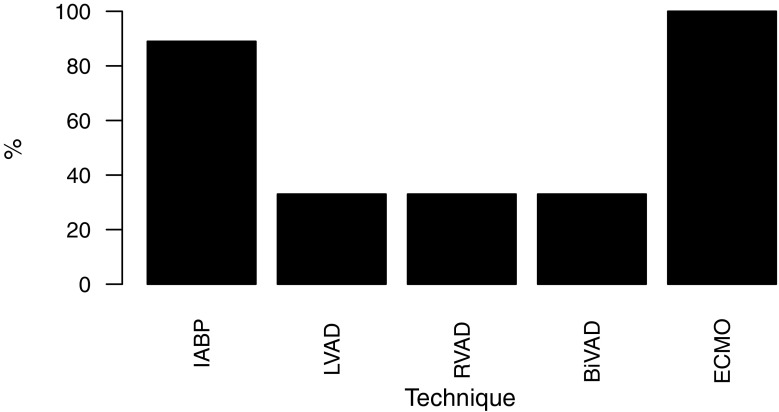
Availability of mechanical cardiac assist devices. *IABP* intra-aortic balloon pump, *LVAD* left ventricular assist device, *RVAD* right ventricular assist device, *BiVAD* biventricular assist devices, *ECMO* veno-arterial and veno-venous extracorporeal membrane oxygenation

As first choice for volume therapy all centers use balanced crystalloid solutions (56% Ringer’s acetate solution, 44% Ringer’s lactate solution). As second choice, 6 centers reported the use of 4% succinylated gelatine, 2 centers use human serum albumin solutions and 1 center uses blood products. Gelatine solutions are never used by 3 centers and 6 centres never use hydroxyethyl starch solutions. The three most important targets for hemodynamic stabilization were optimization of central venous pressure (67%), arterial blood pressure (56%) and echocardiographic cardiac filling (33%). The 3 most often used drugs for the treatment of postoperative low cardiac output syndrome were levosimendan (89%), adrenaline (56%) and dobutamine (56%). For the treatment of systemic inflammatory response syndrome noradrenaline (100%), vasopressin (100%) and hydrocortisone (78%) were used. The 3 most reported vasodilators were nitroglycerin (100%), urapidil (100%) and clonidine (56%). All centers use inhalative vasodilators to treat pulmonary hypertension and six centres have the possibility to use inhalative nitric oxide. The 3 most common drugs to treat pulmonary hypertension were inhalative prostacyclin (78%), inhalative nitric oxide (67%) and intravenous levosimendan (44%). The availability of mechanical cardiac assist devices is shown in Fig. 3. Protocols for the use of vasopressor were established in four centes and for transfusions of blood products in five centres. Interestingly, no center offers preoperative autologous blood donation.

## Discussion

Data from this survey that included all Austrian cardiac surgery centers provide insights into the current state of the postoperative management of these patients. The survey covered the extent of monitoring on the ICU as well as treatment strategies that are completely in line with current recommendations [3–5]. In contrast to Germany and Italy all Austrian centers offering cardiac surgery are public hospitals [4, 5]. The average annual case volume per center is slightly higher than that reported for Italy (722 vs. 617 cardiac procedures) but smaller than in Germany (1460 cardiac procedures) [4, 5]. As in Italy, postoperative intensive care of patients following cardiac surgery in Austria is mainly allocated to anesthesiologists. In contrast, in 29% of all German centers postoperative intensive care is managed solely by cardiac surgeons [4, 5].

According to current German S3 guidelines [1] electrocardiogram (ECG), pulse oximetry, continuous invasive arterial and central venous blood pressure, assessment of the fluid balance and blood gas analysis of arterial and central venous blood are considered standard monitoring for patients after cardiac surgery. Likewise, all nine Austrian centers implemented respective postoperative standards in monitoring. Merely two centers reported not measuring central venous oxygen saturation routinely despite a recommendation in the current German guidelines. The use of neurological monitoring continued to the postoperative period is heterogeneous in Austria. Guidelines recommend the intraoperative use only in certain subgroups, such as heart transplantation, whereas several studies report favorable outcome in general cardiac surgery [9, 10]. Whatever the indication for neuromonitoring during surgery is, continuing intraoperatively instituted and frequently expensive neuromonitoring postoperatively seems to make sense as it may detect potentially hazardous situations in critical patients that can not yet be neurologically evaluated [11]. Although PAC is available in all centers it is employed heterogeneously. Despite the fact that PAC was the only advanced monitoring technique that was associated with reduced mortality in goal-directed therapy [12], its use has been criticized for being related to various risks without actually improving outcome in coronary artery bypass surgery [13]. The current German S3 guidelines [1] name preoperative right heart dysfunction and patients at risk for low cardiac output syndrome and/or pulmonary hypertension as indications for PAC-guided hemodynamic management. This corresponds to the reported indications in this survey. Different frequency in its usage in this study may partly be explained by different risk profiles of patients in the nine centres.

In spite of the high expenses for personnel, training and technical support, all centers can offer transesophageal echocardiography by an experienced user at all times. Kastrup et al. [4] showed that on German intensive care units caring for patients undergoing cardiac surgery in 2005, only 65% had a 24h availability of an experienced user. This lack in manpower or insufficient training may partly be explained by technical and medical progress as in Italy in 2013 [5] all centers had a 24h availability of an experienced user. This survey confirms once more the importance of this technique in the care of patients following cardiac surgery [14].

Despite of the low absolute number of centers in Austria the results of this survey showed remarkable differences in postoperative clinical management of patients undergoing cardiac surgery. In Austria crystalloids are the first choice for volume expansion for all centers. Recent surveys on fluid resuscitation in cardiac surgery in the USA [15] and Europe [6] showed a more diverse picture. For colloids the Austrian results are in line with the European results naming gelatine as the preferred colloid in the majority of the centers [6].

Although controversial, [16] central venous pressure together with arterial blood pressure are in Austria, as in Italy and Germany, the preferred parameters to guide fluid therapy. Echocardiographic filling as additional parameter underlines the importance of echocardiography. In Austria too, dynamic indices derived from additional devices are only infrequently used in routine care to guide fluid management despite evidence of feasibility and accuracy of these devices in this setting [17, 18].

The most important drug to treat low cardiac output syndrome was levosimendan. Shortly after this survey three major trials [19–21] put the expected effect of levosimendan in cardiac surgery into perspective, which prompted a more conservative expert consensus regarding its use in this context [22]. This may have changed the centers’ attitude towards use of this inotrope. There is high accordance regarding employment of systemic vasoconstrictors and vasodilators among all centers. The beneficial effect of aerosolized vasodilators and inhaled nitric oxide for the treatment of pulmonary hypertension has been proven [23]. Although inhalative nitric oxide needs expensive technical equipment it is favored by all centers where it is available as compared to aerosolized vasodilators as potential alternatives.

A limitation of this study is that it is not known if the answers of the selected anesthesiologists truly reflect the actual state at their institution. In this respect, differences in care between different intensivists of the same institution must also be taken in consideration [24]; however, due to local protocols clinical management should be more or less homogeneous within one center. A further limitation of this study is that besides the primary ICU responsible for cardiac surgical patients, some patients go on ICUs managed by other disciplines (e. g. internal medicine) at the same institution that are not covered by this survey. Additionally, this survey did not cover the full scope of postoperative intensive care but only hemodynamic monitoring and treatment of common postoperative disorders.

In conclusion, this is the first investigation providing insights into the current state of postoperative care of cardiac surgical patients in Austria. As proposed by international guidelines standard monitoring is well established and routinely used on Austrian ICUs. Echocardiography is widely seen as a very important tool in the care of cardiac surgical patients and is always available in every center. Use of PAC is heterogeneous with only 33% of centers reporting frequent use. Crystalloids are the fluids of choice in all centers for volume replacement. If needed gelatine solution is the most frequently used colloid. Low cardiac output syndrome is most often treated with levosimendan. Knowledge about the status quo of postoperative intensive care management of cardiac surgical patients allows for further development of patient care. The definition of specific indications for implementation of certain treatment measures, clinical endpoints, and their regular evaluation would be the next steps to systematically improve care after cardiac surgery in Austria on a national base.

## Caption Electronic Supplementary Material

Electronic supplementary material: Original questionnaire [German]
