# Supplementary material for: Postoperative management of patients undergoing cardiac surgery in Austria: A national survey on current clinical practice in hemodynamic monitoring and postoperative management
Source: Wien Klin Wochenschr. 2018 Oct 29;130(23):716–21. doi: 10.1007/s00508-018-1403-3 (PMC6290729; doi:10.1007/s00508-018-1403-3)
Supplement: Supplementary file 1 — Electronic supplementary material: Original questionnaire [German] [file 508_2018_1403_MOESM1_ESM.docx]

Dieser Fragebogen soll den aktuellen Stand beim hämodynamischen Monitoring und der Therapie mit Vasopressoren und Inotropika bei postoperativen herzchirurgischen Patienten in Österreich untersuchen.
Vielen Dank im Voraus für Ihre Teilnahme

Mit freundlichen Grüßen

--------------------

Name:

Name des Krankenhauses:

1. Wie viele Patienten erhielten an ihrer Abteilung im letzten Jahr herzchirurgische Eingriffe:
   1. Anzahl: ______
   2. Anzahl mit Herz-Lungen-Maschine: _____
   3. Patienten mit dringlichen Eingriffen in Prozent ____%
   4. Pateinten mit Notfalleingriffen in Prozent ____%
   5. Patienten mit Re-Do Eingriffen in Prozent ____%
2. Bitte geben Sie die Anzahl der Patienten an, die folgende Eingriffe an Ihrer Einrichtung erhielten:
   1. CABG ______
   2. Aortenklappenchirurgie ______
   3. Mitralklappenchirurgie ______
   4. Aortenchirurgie ______
   5. Kombinationseingriffe ______
   6. Permanente Herzunterstützungssysteme (LVAD/RVAD) ______
   7. Herztransplantationen ______
   8. Sonstige ______
3. Wie viele Kinder erhielten im letzten Jahr Herzchirurgische Eingriffe an Ihrer Einrichtung?
   1. Anzahl:_____
   2. Anzahl mit Herz-Lungen-Maschine: _____
4. Die postoperative intensivmedizinische Betreuung erwachsener herzchirurgischer Patienten erfolgt durch: (mehrfache Auswahl möglich)
   1. Anästhesisten
   2. Herzchirurgen
   3. Internisten
5. Die Intensivstation für die postoperative Betreuung herzchirurgischer Patienten untersteht:
   1. Abteilung für Anästhesie
   2. Abteilung für Chirurgie
   3. Abteilung für Innere Medizin
6. Die Intensivstation für die postoperative Betreuung herzchirurgischer Patienten ist:
   1. nur für herz-/thoraxchirurgische Patienten
   2. eine gemischte Intensivstation
7. Welches Monitoring wird routinemäßig bei herzchirurgischen Patienten auf Ihrer Intensivstation eingesetzt? (bitte verwenden Sie folgende Wörter: immer, manchmal, selten, nie)
   1. EKG ______
   2. invasiver arterieller Blutdruck ______
   3. ZVD ______
   4. Linksatriale Druckmessung ______
   5. SaO2 ______
   6. ScO2 ______
   7. SpO2 ______
   8. etCO2 ______
   9. EEG (inkl. BIS) ______
   10. NIRS (z.B. INVOS) ______
   11. Stundenharnmengen ______
   12. Temperatur ______
8. Welches erweitertes hämodynamische Monitoring ist auf ihrer Intensivstation verfügbar?
   1. Pulmonaliskatheter
   2. PiCCO- System
   3. Vigileo- System
   4. LiDCO- System
   5. TEE
   6. TTE
   7. Andere
9. Wenn Sie erweitertes hämodynamisches Monitoring verwenden, erläutern Sie bitte die Häufigkeit (bitte verwenden Sie folgende Wörter: häufig, manchmal, selten, nie).
   1. Pulmonaliskatheter ______
   2. PiCCO- System ______
   3. Vigileo- System ______
   4. LiDCO- System ______
   5. TEE ______
   6. TTE ______
   7. Andere (bitte erläutern) ______
10. Wenn Sie den Pulmonaliskatheter verwenden, was sind ihre Indikationen? (Bitte geben Sie Ihre erste, zweite und dritte Wahl an)
    1. Monitoring von hämodynamischer Instabilität
    2. Messung des Herzzeitvolumens
       1. Falls ja, ab welcher EF: ______
    3. Monitoring des systemischen Gefäßwiederstandes (SVR)
    4. Management von Volumentherapie
    5. Monitoring von inotroper/ vasopressor Therapie
    6. Monitoring von Svo2
    7. Monitoring im septischen Schock
    8. Monitoring von pulmonaler Hypertension
       1. Falls ja, ab welchem präoperativen sysPAP ______
11. Falls Sie Messungen mit dem Pulmonaliskatheter durchführen, sind die Messungen:
    1. kontinuierliche HZV Messungen
    2. intermittierende HZV Messungen
       1. Falls sie beide Systeme verwenden geben Sie ungefähr den Anteil der kontinuierleichen Messung an: ______%
12. Ist ein qualifizierter Arzt rund um die Uhr verfügbar um eine TEE Untersuchung durchzuführen?
    1. Ja
    2. Nein
13. Was sind Ihre Indikationen ein TEE durchzuführen? (Bitte geben Sie Ihre erste, zweite und dritte Wahl an)
    1. hämodynamische Instabilität
    2. Verdacht auf Tamponade
    3. Verdacht auf thrombembolische Events
    4. Verdacht auf regionale Wandbewegungsstörungen
    5. Management von Volumentherapie
    6. Bewertung der Kalppenfunktion
14. Bitte geben Sie die erste, zweite und dritte Wahl für Volumentherapie im OP und auf der Intensivstation an
    1. Erste Wahl: _____
    2. Zweite Wahl: _____
    3. Dritte Wahl: _____
15. Haben Sie Dosisbeschränkungen für den Gebrauch von Gelatine-Lösungen auf Ihrer Intensivstation
    1. Wir verwenden keine/ extrem selten Gelatinelösungen
    2. Nein
    3. Ja (bitte erläutern) ______
16. Haben Sie Dosisbeschränkungen für den Gebrauch von Stärke-Lösungen auf Ihrer Intensivstation
    1. Wir verwenden keine/extrem selten Stärkelösungen
    2. Nein
    3. Ja (bitte erläutern) ______
17. Welche Albuminlösungen verwenden Sie in Ihrer auf Ihrer Intensivstation
    1. Albumin 3,5%
    2. Albumin 20%
    3. Albumin 5%
    4. Wir verwenden keine/extrem selten Albuminlösungen
18. Welche hämodynamische Zielgrößen verwenden Sie für die Steuerung der Flüssigkeitstherapie?
    1. Erste Wahl ______
    2. Zweite Wahl ______
    3. Dritte Wahl ______
19. Um ihre Zielgrößen zu erreichen verwenden Sie eher einen Flüssigkeitbolus mit
    1. Kristalloiden
    2. Kolloiden
20. Welche Medikamente verwenden Sie für die Behandlung des LCOS (low cardiac output syndrome)? (Bitte geben Sie Ihre erste, zweite und dritte Wahl an)
    1. Adrenalin
    2. Noradrenalin
    3. Dopamin
    4. Dobutamin
    5. Levosimendan
    6. Milrinon
    7. Andere
21. Welche Kombination vasoaktiver Substanzen verwenden Sie für die Behandlung des LCOS (Bitte auflisten)
    1. Erste Wahl ______
    2. Zweite Wahl ______
    3. Dritte Wahl ______
22. Welche vasoaktiven Medikamente verwenden Sie in der Behandlung des post-perfusions Syndroms /postoperatives SIRS
    1. Noradrenalin
    2. Vasopressin
    3. Hydrocortison
    4. Andere (bitte erläutern)
23. Welche inotropen oder vasoaktiven Substanzen verwenden Sie für die Therapie des Rechtsherzversagens? (Bitte auflisten)
    1. Erste Wahl ______
    2. Zweite Wahl ______
    3. Dritte Wahl ______
24. Welche Vasodilatoren werden auf Ihrer Intensivstation verwendet? (Bitte geben Sie Ihre erste, zweite und dritte Wahl an)
    1. Nitroglycerin
    2. Natrium-Nitroprussid
    3. Uradipil
    4. Clonidin
    5. Prostacyclin
    6. Prostaglandin E
25. Verwenden Sie inhalative Vasodilatoren auf Ihrer Intensivstation?
    1. Ja
    2. Nein
26. Verwenden Sie ein NO Gerät zur Behandlung von schwerer pulmonaler Hypertension?
    1. Ja
    2. Nein
27. Welche vasoaktiven Medikamente verwenden Sie beim Management der schweren pulmonalen Hypertension?
    1. Erste Wahl ______
    2. Zweite Wahl ______
    3. Dritte Wahl ______
28. Haben Sie ein Protokoll für die Anwendung Vasoaktiver Substanzen auf Ihrer Intensivstation?
    1. Ja
    2. Nein
29. Welche mechanischen Assist Devices sind auf Ihrer Intensivstation verfügbar
    1. IABP
    2. LVAD
    3. RVAD
    4. BIVAD
    5. V-A ECMO
    6. V-V ECMO
    7. Keine
    8. Andere (bitte erläutern) ______
30. Verfügt Ihre Intensivstation über ein Transfusionsprotokoll?
    1. Ja
    2. Nein
31. Gibt es an Ihrer Klinik ein Programm zur Eigenblutspende für herzchirurgische Patienten?
    1. Ja
       1. Falls ja, wieviel Prozent der Patienten nimmt es in Anspruch?
    2. Nein
